# Pooled testing with replication as a mass testing strategy for the COVID-19 pandemics

**DOI:** 10.1038/s41598-021-83104-4

**Published:** 2021-02-10

**Authors:** Julius Žilinskas, Algirdas Lančinskas, Mario R. Guarracino

**Affiliations:** 1 Institute of Data Science and Digital Technologies, Vilnius University, Vilnius, Lithuania; 2 HSE – National Research University Higher School of Economics, LATNA Laboratory, Nizhny Novgorod, Russia; 3 University of Cassino and Southern Lazio, Cassino, Italy

**Keywords:** Viral infection, Sequencing, Applied mathematics

## Abstract

During the COVID-19 pandemic it is essential to test as many people as possible, in order to detect early outbreaks of the infection. Present testing solutions are based on the extraction of RNA from patients using oropharyngeal and nasopharyngeal swabs, and then testing with real-time PCR for the presence of specific RNA filaments identifying the virus. This approach is limited by the availability of reactants, trained technicians and laboratories. One of the ways to speed up the testing procedures is a group testing, where the swabs of multiple patients are grouped together and tested. In this paper we propose to use the group testing technique in conjunction with an advanced replication scheme in which each patient is allocated in two or more groups to reduce the total numbers of tests and to allow testing of even larger numbers of people. Under mild assumptions, a 13 ×  average reduction of tests can be achieved compared to individual testing without delay in time.

## Introduction

The COVID-19 pandemic has already affected millions of people among more than 200 countries around the world. The incidence of the disease in different countries varies from several to hundreds of positive cases per 1000 tests (data retrieved on December 14th, 2020 from https://www.worldometers.info/coronavirus/).

The fast spread of the virus made it clear that in our globalised world threats can spread fast. The lack of knowledge related to the virus, the contrasting information available, the absence of a targeted vaccine and healing procedures made it soon clear that the spread could be mitigated only with restrictions on personal mobility. The quarantine and personal isolation have been applied nearly worldwide. Such restrictions can be made both less stringent and more effective if tests can early detect the presence of infectious individuals. The scarcity of reactants, accredited laboratories for virus testing, and trained technicians have so far severely limited the number of tests.

The available tests are based on oropharyngeal and nasopharyngeal swabs and the collected organic material. The virus is then inactivated, and RNA is extracted and amplified. A real-time polymerase chain reaction (RT-PCR) is then used to detect and evaluate the abundance of proteins that are virus-specific. In order to test as many people as possible, studies have shown the possibility of group testing^1^, which means that the result of the test is given for a group of people, rather than for a single patient. In the simplest case, this might be applicable to people living in the same apartment, or sharing the same working spaces. In the case of an infected person in a group, the test result for the group is positive, and additional tests are needed.

Experiments indicate that a single positive sample of COVID-19 can be detected in pools of up to 32 samples, with an estimated false negative rate of 10%, and detection of positive samples diluted in even up to 64 samples may also be attainable, though may require additional amplification cycles^2^.

Other investigations^3^ indicate that a mini-pool method can obtain laboratory results of equal quality as those obtained with individual testing. The method was investigated in a small field study on 50 unselected patient samples. Mini-pools of 5 samples were tested, all mini-pools with a positive sample resulted in a positive PCR result, and all mini-pools containing only samples from patients without SARS CoV-2 always resulted in a negative result.

This enables pool testing for COVID-19 where the swabs of multiple patients are grouped together and tested. If the result of a sample pool is negative, all the samples in the group are negative. In the case of a positive pool result, individual testing is carried out from the previously reserved samples. Depending on the incidence of the disease in the population, only a fraction of the groups will need to be tested again for individual results, and therefore the number of tests is reduced accordingly.

For example, suppose 96 samples should be tested and pools of up to 12 samples are possible. In individual testing, 96 tests are necessary. In pool testing, 8 pools of 12 samples are composed and testing is performed. Imagine, that the result of one pool is positive so additional 12 individual tests are needed. Therefore, 20 tests are performed instead of 96. So the number of tests is decreased approximately 5 times with the same number of samples tested. Approximately 5 times more samples could be tested with the same resources. The procedure becomes less efficient when there could be more than one positive sample, e.g. in the case of 3 positive samples in different pools, $8+12\times 3 = 44$ tests would be required. On the other hand, if 12 pools of 8 samples were used and there were 3 positive samples in different pools, $12+8\times 3 = 36$ tests are required. Therefore, the pool size could be optimized depending on the incidence. A smaller incidence enables the use of larger pools. In addition to optimization of the pool size, replication of samples in different pools may help identify infected individuals by analysing intersecting elements among positive pools. The replication increases the number of pool tests, but may significantly reduce the number of individual tests. See for example transposition based replication^4^ there in after.

Group testing can be optimised to multiply the power of tests against COVID-19^5,6^. For an incidence of 20 positive samples per 1000 tested, the groups of size 80 are optimal from the statistical point of view^5^. In practice, technical limitations as well as the cost of grouping the individual samples put a downwards pressure on the group size. For an incidence of 100 positive samples per 1000, 40.6% of tests can be saved using testing groups of four subjects, for an incidence of 200, 17.9% of tests can be saved using groups of three subjects^6^.

Non-adaptive and adaptive group testing strategies based on the binary splitting is proposed in^1^. The method starts by choosing a group from the population to be tested, performing a test on the combined sample from the entire group and progressively splitting the group further into subgroups in the case of positive results. Such a hierarchical strategy requires several stages and an extra time. The patients with symptoms are individually tested, and the pooling is applied only to the asymptomatic ones, in order to reduce the number of positive pools needing the binary search.

The group testing is not a new concept and was used in identification of other diseases to make testing timely and cost effective, see for example^7^ and references therein. Many previously proposed strategies may be adapted to the group testing of COVID-19.

In our previous work^8^ we proposed optimization of pooled experiments of Next Generation Sequencing for detection of rare mutations. In the study the main aim was to reduce costs and this was achieved by replication of patients in the pools. The proposed replication strategies included transposition and OptReplica. A web-oriented software for the optimization of pooled experiments^9^ may be used for optimal planning of pools and replications. A similar idea for replication of patients may be performed in COVID-19 testing but here limited resources are more important than the price.

## Methods

This paper addresses two concepts which are important for a mass testing: grouping of samples and their replication among groups. The research is aimed at investigation of a new strategy for grouping patients into pools with replication for pooled testing for COVID-19 in order to highlight its strengths and limitation as well as possible use cases.

### Group testing

Pooling of samples and the distribution into multiple pools were evaluated in^10^, where 384 patient samples were pooled into 48 pools of 48 samples with each sample distributed to six pools. The strategy provides 8-fold increase in testing efficiency. Five sets of 384 samples, containing 1–5 positive carriers were screened and all positive carriers in each set of experiments were correctly identified.

Group testing for COVID-19 is evaluated in^4^ where the testing procedure exactly corresponds to pool testing with transposition based replication^8^. When most tests are negative, pooling reduces the total number of tests up to four-fold at 2% incidence and eight-fold at 0.5% incidence.

### Replication of samples

The replication of samples into pools is performed in such a way that it is possible to directly identify a single positive sample from the positive pools. It is worth to perform replication in mass testing of asymptomatic persons, while patients with symptoms are individually tested. We assume up to 32 samples in a pool, but the largest pool size may be adjusted depending on the reliability of tests.

**Figure 1:**
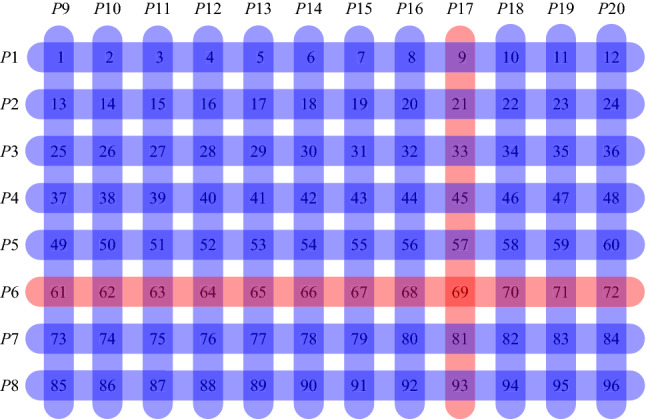
Transposition based replication.

Let us start from the illustration of the transposition based replication using the example of 96 patients and available pooling of 12 samples. When the transposition replication is used, apart from 8 main pools, 12 control/replicated pools are formed with different compositions of samples. One can imagine an $8\times 12$ array of samples and the main pools may correspond to the rows of samples and the control pools then correspond to the columns of samples. This is illustrated in Fig. 1. By performing 20 tests (8 main pools and 12 control pools) a single positive sample may be indicated by the positive result of one specific main and one control pools. So the same 20 number of tests, the same resources as using simple pooling, but the shorter time because of an unnecessary two-step procedure. In the case of 3 positive samples in different pools, additional individual tests would be necessary with the maximal total amount of tests being 29 compared to 44 or 36 tests of simple pooling illustrated in the previous section.

Transposition based replication is not the best option when the size and number of pools is different and therefore it is limited when the optimal size of pool is searched. Our proposed OptReplica strategy enables a better grouping of patients into pools. Each patient is allocated in the first pool that is not yet completely filled, and replicated in the first pool with the smallest number of allocated patients. Such an allocation of patients guarantees that the sample and its replica will be in different pools and the logical conjunction of two different pools is the only sample.

**Figure 2:**
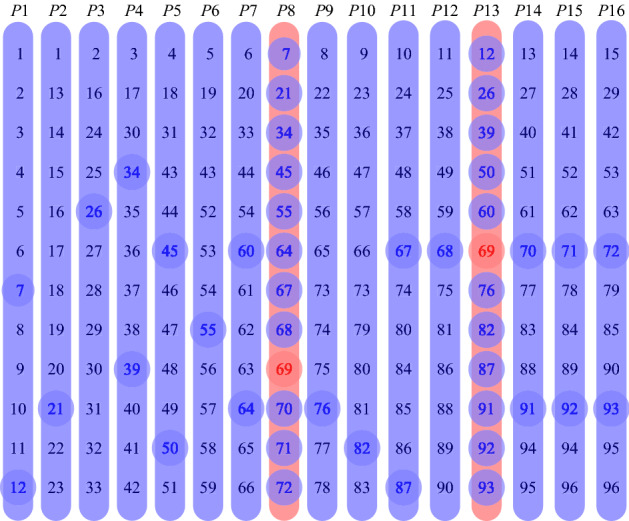
OptReplica replication.

For example, 96 patients may be allocated to 16 pools with 12 samples each and therefore requiring 16 tests to indicate one positive sample out of 96. The allocation and indication are illustrated in Fig. 2. The number of 16 tests is smaller than 20 tests in simple pooling or the same 20 tests in the transposition based replication.

For OptReplica to work, the number of pools (*p*) must be larger than the size (*m*) of the largest pool, $p > m$. This implies that the maximal pool size is the largest number *m*, which satisfies the following inequality:

1 $$\begin{aligned} m \times (m + 1) \le 2n. \end{aligned}$$

Then the number of pools is:

2 $$\begin{aligned} p = \left\lceil {\frac{2n}{m}} \right\rceil . \end{aligned}$$

After pooling of samples with OptReplica, all pools are tested. If two pools are positive and the remaining pools are negative, then the sample conjugating the positive pools is positive, and the remaining samples in that two pools are negative together with all samples in negative pools.

If more than two pools are positive, then all patients, whose samples and their replicas belong to these positive pools must be considered as suspects and individual tests must be performed to validate or negotiate the disease. The number of individual tests can be reduced by analyzing conjunctions of the positive pools; e.g., if we have four positive pools and they are conjugated in pairs, then those two conjugating samples are definitely positive.

Depending on the probability of positive samples within the target population and the number of possible samples in a pool, optimal allocation of pools may be found.

### Experimental investigation

The experimental investigation based on simulation modelling and analysis was performed to investigate applicability of the proposed strategy to efficient testing for COVID-19 and compare its efficiency with transposition based replication which application to testing for COVID-19 was investigated in^4^. It was assumed that two populations of $n = 96$ and $n = 384$ samples are subject to be tested considering different incidences of the disease, varying from 1 to 100 cases per 1000 tests. To simulate different incidences of the disease, each sample in a population was considered as infected with an appropriate probability; for example, to simulate 10 positive cases per 1000 tests, each sample is considered as infected with probability 0.01. Due to the randomness, each experiment has been run for 100 times using randomly generated populations and average results were analysed.

All samples were grouped into pools with replication and the number of group tests and the number of single tests required to determine infected samples were counted. Different pool sizes which divide all samples and their replicas into completely filled pools and the largest possible pool size were investigated in order to find the dependency of the efficiency of a strategy on the pool size.

The efficiency of a pooling strategy were measured by savings of tests (in times) using a pooling strategy comparing with the individual testing of a target population. Assuming that the testing of a population of *n* individuals without pooling requires *n* tests and the testing of the same population using the strategy *S* requires *T*(*S*) tests, the efficiency of the strategy *S* can be mathematically expressed by

3 $$\begin{aligned} E(S) = \frac{n}{T(S)}. \end{aligned}$$

In the case of individual testing which requires to test all samples, $E(S) = 1$. If a strategy *S* requires two times less tests than the individual testing, then $E(S) = 2$. A larger efficiency value means a better efficiency of the strategy.

In this paper we focus on the worst case analysis assuming that all patients, whose samples and their replicas belong to positive pools, must be tested individually, except the situation when only two pools are positive.

In the future, we will address the problem of different contribution of organic material from different patients pooled together. In order to optimize the pool size with respect to both incidence and sampled proportion, we will extend the results obtained in a previous work^11^.

## Results

Two populations of 96 and 384 individuals have been used in order to compare obtained results with those given in^4^. The experimental investigation of the transposition based replication using 8 columns by 12 rows pooling of 96 samples and 16 columns by 24 rows pooling of 384 patients gave very similar results to those published in^4^.

**Figure 3:**
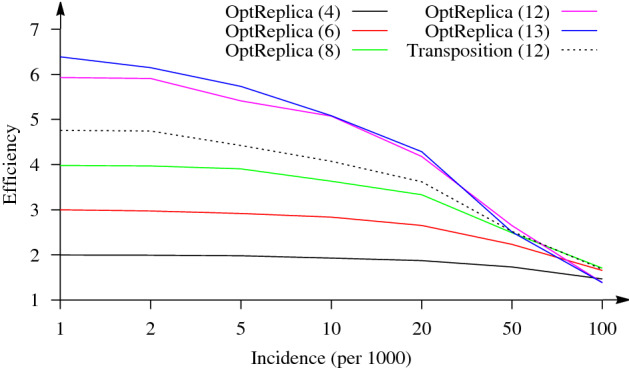
Dependence of the efficiency of OptReplica with different pool sizes and the transposition based replication on the incidence of the disease testing 96 samples.

Several pool sizes ($m \in \{4, 6, 8, 12\}$) which divide $2 \times 96 = 192$ samples into completely filled pools and the largest possible pool size ($m = 13$) for replication of 96 samples were selected for OptReplica. Results of all experiments are presented in Table 1. The comparison of the efficiency in testing 96 samples using the transposition based replication and OptReplica is illustrated in Fig. 3. Here the horizontal axis stands for the incidence of the disease and the vertical axis—for the efficiency of a pooling strategy. The dashed curve indicates the efficiency of the transposition based replication and continuous curves indicate the efficiency of OptReplica with the selected pool sizes.

One can see from the figure that the replication strategy OptReplica can reduce the number of tests needed to determine infected patients in the population of 96 individuals by more than 6 times when the incidence is low. In comparison, using the transposition based replication the number of tests can be reduced by less than 5 times in the case of the same incidence. The best results with OptReplica were achieved using large pools (up to 12 and 13 samples per pool) when the incidence is lower than 50 cases per 1000 tests. In the case of a larger incidence, it is better to use smaller pools (8 samples per pool) for OptReplica.

**Table 1:** Efficiency of OptReplica and the transposition based replication testing 96 samples.

Pooling	*p*	*m*	Incidence (per 1000)						
			1	2	5	10	20	50	100
OptReplica	48	4	2.00	2.00	1.99	1.93	1.86	1.73	1.47
	32	6	3.00	2.97	2.92	2.84	2.65	2.23	1.66
	24	8	3.99	3.97	3.91	3.63	3.33	2.488	**1.71**
	16	12	5.93	5.91	5.41	5.06	4.18	**2.65**	1.39
	15	13	**6.39**	**6.15**	**5.73**	**5.08**	**4.29**	2.51	1.38
Transposition.	8	12	4.76	4.75	4.43	4.07	3.62	2.52	1.68

The best results for each incidence are marked in bold.

**Figure 4:**
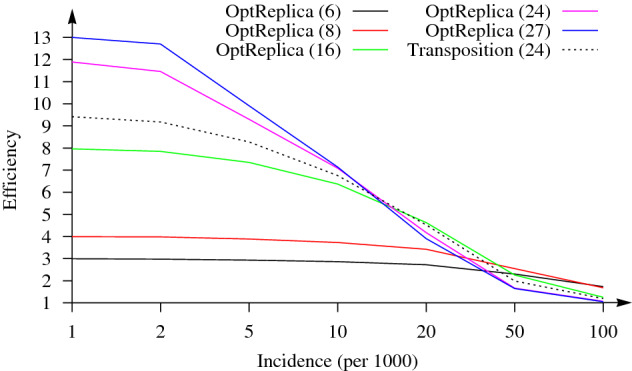
Dependence of the efficiency of OptReplica with different pool sizes and the transposition based replication on the incidence of the disease testing 384 samples.

**Figure 5:**
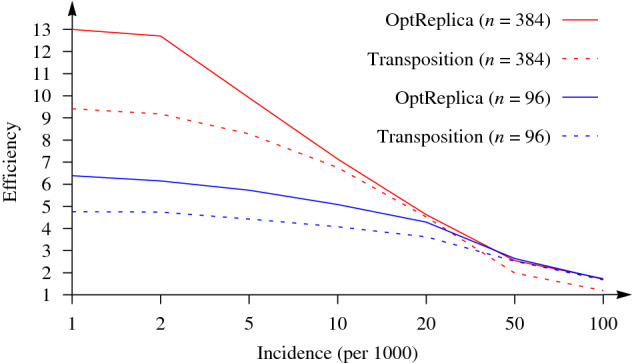
Efficiency of OptReplica with the optimal pools sizes versus the transposition based replication.

Similar results have been obtained with $n=384$ patients. Here, pool sizes ($m \in \{4, 6, 8, 12, 16, 24\}$) which divide $2 \times 384 = 768$ samples into completely filled pools and the largest possible pool size ($m = 27$) for replication of 384 samples were selected for OptReplica. Results of all experiments are presented in Table 2. Selected results are illustrated in Fig. 4, from which we can see that the replication strategy OptReplica can reduce the number of tests needed to determine infected patients by around 13 times when the incidence is small. Figure 5 illustrates comparison of the efficiency of OptReplica with the optimal pool size, chosen depending on the incidence, with efficiency of the transposition base replication.

**Table 2:** Efficiency of OptReplica and the transposition based replication testing 384 samples.

Pooling	*p*	*m*	Incidence (per 1000)						
			1	2	5	10	20	50	100
OptReplica	192	4	2.00	1.99	1.98	1.95	1.90	1.74	1.50
	128	6	2.99	2.98	2.93	2.86	2.73	2.30	**1.74**
	96	8	3.99	3.98	3.88	3.73	3.42	**2.55**	1.68
	64	12	5.97	5.95	5.59	5.17	4.32	2.53	1.47
	48	16	7.96	7.85	7.35	6.37	**4.63**	2.26	1.26
	32	24	11.89	11.46	9.30	7.09	4.17	1.66	1.07
	29	27	**13.00**	**12.70**	**9.91**	**7.14**	3.90	1.65	1.05
Transposition	16	24	9.41	9.17	8.28	6.76	4.52	1.99	1.19

The best results for each incidence vale are marked in bold.

The results of the experimental investigation presented in Figs. 3 and 4 showed that OptReplica can effectively determine positive samples with using more than 13 times less tests, comparing with individual testing when all patients are tested. Although, the efficiency of the proposed strategy depends on the incidence of the disease, choosing the right pool size makes OptReplica better than transposition based replica independent on the incidence. The optimal pools for testing 96 and 384 with respect to the incidence of the disease is presented in Table 3.

**Table 3:** Optimal pool sizes for OptReplica with respect to the incidence of the disease.

*n*	Incidence (per 1000)						
	1	2	5	10	20	50	100
96	13	13	13	13	13	12	8
384	27	27	27	27	16	8	6

## Discussion

The experimental investigation showed that the proposed strategy for pooling with replication OptReplica can reduce the number of tests more than up to 13 times comparing with individual testing strategy. The efficiency of the strategy depends on the pool size which varies depending on the incidence of the disease. If the ratio of positive tests is 10 or less per 1000 tests, then it is worth to use the maximal possible pool size. If the incidence is higher then lower pool sizes should be used, and in the case of the incidence of 50 cases per 1000 samples or larger, it is worth to consider using other strategies for pooling with replication or strategies with pooling without replication.

The efficiency of the proposed strategy OptReplica is notably better than the efficiency of the transposition based replication in the case of the same incidence. Smaller pools must be chosen for OptReplica when the incidence reaches 10–20 positive tests out of 1000. However, even when the incidence is low, choosing the right pool sizes OptReplica demonstrated notably better efficiency than the transposition based replication by the same incidence. The obtained results show that independent on the incidence of the disease OptReplica in average gives better efficiency than the transposition based replication if the right pool size is used: larger pools if the incidence is smaller and smaller pools, if the incidence is larger.

The proposed strategy OptReplica can be useful in situations where chance to detect a disease is low, e.g. testing of population of a region without known outbreaks of the virus or assumptions for high incidence, performing control tests for recovered patients, etc.

Due to specific allocation of samples and their replicas among pools OptReplica has limited pool size, which is 13 when testing population of 96 samples and 27 when testing 384 samples. On the other hand it should be reasonable taking into account other limitations for mixing samples.

The proposed strategy suggests to perform individual testing of patients assigned to positive pools except occurrence of two positive pools. However, it is possible to identify infected individuals by analysing intersections of more than two positive pools. Such an improvement of the proposed strategy will be an object of our further research.

## Conclusions

In this paper we proposed a testing scheme with pools and replication to detect patients positive to COVID-19. We provide examples on how to allocate patients in pools with a strategy that is optimal and minimizes the number of required tests. We show that for different levels of incidence, it is always possible to find a grouping of the patients that minimizes the number of tests. Our strategy outperforms other techniques, and can reduce the overall number of tests up to 13 times on average.

## Human data

No human data nor samples were used for this study.
